# Early-Phase Response of Broiler Breeders to 25-Hydroxyvitamin D_3_ and Canthaxanthin with or Without Copper and Gluco-Oligosaccharides (30 to 41 Weeks)

**DOI:** 10.3390/ani16121848

**Published:** 2026-06-15

**Authors:** Patrick Tamatey, John W. Boney, Dervan D. L. S. Bryan

**Affiliations:** Department of Animal Science, The Pennsylvania State University, University Park, PA 16802, USA; pxt5221@psu.edu (P.T.);

**Keywords:** broiler breeders, egg quality, fertility, canthaxanthin, 25-hydroxyvitamin D_3_

## Abstract

This study evaluated the effects of dietary supplementation with 25-hydroxyvitamin D_3_ (25-OH-D3), canthaxanthin (Cx), copper (Cu), and gluco-oligosaccharides (GO) on performance, egg quality, fertility, and hatchability of broiler breeder hens during early production (30 to 41 weeks of age). A total of 210 breeder hens and 21 males were allocated to three dietary treatments. Birds were fed a standard broiler breeder diet (control) or the same diet in which 0.5 kg/MT of an additive premix replaced sand. This premix supplied 16.6–17.7 mg/kg canthaxanthin (Cx) and 3700–4700 IU/kg 25-OH-D3 in Treatment 1, and 9.2–11.1 mg/kg Cx and 4100–4700 IU/kg 25-OH-D3 in Treatment 2. Treatment 2 also included Cu and GO (≥44 mg/g within the additive), with Cu provided at industry-standard levels. Each treatment consisted of seven replicates, with birds housed in floor pens containing 10 females and 1 male per replicate. Hen performance was recorded weekly, while egg quality was assessed at 30, 35, and 41 weeks. Fertility and hatchability were evaluated at 30 and 41 weeks. Treatment 2 improved lay rate, albumen height, Haugh unit, and feed conversion ratio (FCR) at selected time points. Both supplemented diets consistently produced darker yolk color compared with the control. Supplementation consistently enhanced yolk color, whereas effects on albumen height, Haugh unit, shell thickness, and FCR were observed only at specific ages or weeks. Lay rate differed among treatments only at week 40, with a trend observed at week 41. Fertility and hatchability were not significantly affected by dietary treatment.

## Simple Summary

Broiler breeder hens must produce high-quality eggs for efficient poultry production, yet egg quality and reproductive performance often decline with age. This study evaluated whether dietary supplementation with 25-hydroxyvitamin D_3_ and canthaxanthin, with or without copper and gluco-oligosaccharides, could improve early lay performance. Supplementation consistently enhanced yolk color and improved some internal egg quality and efficiency variables at specific ages or weeks; however, these responses were not sustained across the entire early production period. Fertility and hatchability were not significantly affected during the early production phase.

## 1. Introduction

Broiler breeders are important in the poultry industry as the parent stock responsible for producing fertile eggs used to generate broilers for meat production. The United States produces approximately 20% of global broiler meat production. In 2022, the USA, together with Canada and Mexico, produced 20,652,970.9 tons of broiler meat [1]. The breeder industry contributes to food security because the broilers they produce remain one of the leading animal protein sources [2]. Broiler breeders are essential for ongoing research, allowing for advancements in nutrition, health, and management practices that benefit the entire poultry industry. However, reproductive efficiency in broiler breeders has not shown comparable improvement [3]. Broiler hatchability in the United States began to drop suddenly in 2010 after years of sustained improvements and has not shown signs of recovery [3]. The decline in hatchability has resulted in financial losses, including an annual loss of over 550 million potential broilers, accounting for approximately 6% of the industry’s return [4]. Despite the severity of the issue, the reasons behind this trend remain unclear, prompting investigation into nutritional strategies such as supplementation with 25-OH-D3 and Cx, which may influence oxidative status, nutrient utilization, and reproductive performance in broiler breeders [5]. As broiler breeders age, declines in productive and reproductive performance have been widely reported [5,6]. One contributing mechanism is oxidative stress, which disrupts cellular function by imbalancing the production of reactive oxygen species (ROS) and antioxidant defense systems [7]. This imbalance can impair physiological processes critical for egg production and embryo development [8]. Nutritional strategies targeting oxidative stress, particularly the use of antioxidants such as carotenoids and 25-OH-D3, have been shown to enhance antioxidant capacity in broiler breeders and may help mitigate age-related declines in performance [8]. Gluco-oligosaccharides are non-digestible carbohydrates that promote beneficial gut microbiota and increase short-chain fatty acid (SCFA) production in the gastrointestinal tract thereby improving intestinal integrity and enhancing the absorption of key nutrients such as calcium, phosphorus, and vitamins, which are critical for egg formation and embryonic development. Egg quality is a key determinant of reproductive efficiency in broiler breeders, as it directly influences hatchability [9]. Because egg formation relies on adequate nutrient availability, mineral metabolism, and gut health, impairment in gastrointestinal function can negatively affect albumen quality, shell strength, and embryo viability. Therefore, dietary additives that support gut health and nutrient utilization may help maintain egg quality and reproductive performance [10]; however, information on the combined effects of 25-OH-D_3_ and canthaxanthin, with or without Cu and GO, during the early production phase of broiler breeders remains limited. It was hypothesized that dietary inclusion of 25-OH-D_3_, Cx, Cu, and GO would improve breeder hen performance, egg quality, fertility, and hatchability. The objective of this study was to evaluate the effects of these dietary combinations on breeder performance and egg quality from 30 to 41 weeks of age.

## 2. Materials and Methods

### 2.1. Ethical Approval

The Pennsylvania State University Institutional Animal Care and Use Committee (Protocol no. PROTO202402828) approved all procedures.

### 2.2. Birds and Management

Broiler breeder females (Aviagen Ross 708) and males (Hubbard M99) were reared from day-old chicks to 25 weeks of age in separate deep-litter houses following the recommendations of the Ross 708 Parent Stock Commercial Guide and the Hubbard Management Guide. At 25 weeks, 231 birds were randomly assigned to 21 pens in one wing of the broiler research barn with dimensions 14.6 m × 7 m × 2.43 m. The research wing had 24 pens, each 2.13 m × 1.14 m. Each pen contained 1 male and 10 females. Wood shavings (12 to 15 cm thick) and a 2-compartment roll nesting box measuring 50.8 × 52.1 × 51.8 cm (Homestead Essentials—Ravenna, OH, USA) were placed into each pen. Environmental conditions in the research barn were monitored and regulated using a MAXIMUS Controller System (MAXIMUS Solutions Inc., Saint-Bruno-de-Montarville, QC, Canada). From day 1 of age, broiler breeder chicks were brooded under a photoperiod of 23 h light: 1 h dark for the first 2 days after placement, as recommended in the Ross Parent Stock Management Handbook. Thereafter, day length was gradually reduced to a short-day rearing program and maintained until photo stimulation at >21 weeks of age, in accordance with Ross 708 parent stock protocols. Each bird was individually weighed at 30 weeks of age at the start of the experiment. Weighing was conducted using an Adam Equipment GBK 70a bench scale (Model GBK 130a; Adam Equipment Inc., Oxford, CT, USA)). Birds were placed in a 12-gallon Sterilite hinged-lid tote (55.3 × 39.1 × 31.8 cm), and body weight was recorded in kilograms.

### 2.3. Diets and Experimental Design

Three dietary treatments were evaluated using a common basal diet. The control diet consisted of the basal formulation supplemented with 0.5 kg/MT sand as an inert filler. Treatment 1 was formulated by replacing the sand with 0.5 kg/MT of an additive blend containing 25-hydroxyvitamin D_3_ (25-OH-D_3_) and canthaxanthin (Table 1). Treatment 2 was formulated similarly but included 0.5 kg/MT of an additive blend containing 25-OH-D_3_, Cx, Cu, and GO. From the analyzed values (Table 2), the diets provided 16.6–17.5 mg/kg canthaxanthin in Treatment 1 and 9.2–11.1 mg/kg in Treatment 2, while 25-OH-D_3_ ranged from 3700 to 4700 IU/kg in both supplemented treatments. Gluco-oligosaccharides were supplied as part of the additive at a minimum concentration of 44 mg/g, and copper was included through a commercial premix at industry-standard levels. All additive blends were incorporated at a fixed inclusion rate of 0.05% (0.5 kg/MT) of the diet. Canthaxanthin and 25-OH-D_3_ concentrations in experimental diets were analytically determined on an as-is basis by Technical Marketing Analytical Services (Belvidere, NJ, USA), and the results are summarized in Table 2. Gluco-oligosaccharides were supplied as a caramel-based product containing a minimum of 44 mg/g gluco-oligosaccharides. Phytase was supplied as HiPhorius^®^ 2400 (DSM Nutritional Products, Parsippany, NJ, USA), a phytase preparation derived from *Aspergillus oryzae* with an activity of 1,099,526–2,424,000 FYT. Although Treatment 1 and Treatment 2 were both formulated at the same additive inclusion rate of 0.05% of the diet, the analyzed canthaxanthin concentration differed between the two supplemented diets by 6.9 mg/kg. Therefore, Treatment 2 differed from Treatment 1 not only by the inclusion of Cu and gluco-oligosaccharides, but also by a lower analyzed canthaxanthin concentration. As a result, direct comparisons between Treatment 1 and Treatment 2 were interpreted as comparisons between two commercial additive blends rather than as an isolated test of Cu and gluco-oligosaccharide effects. Each diet was fed to seven replicate pens per treatment, with pen serving as the experimental unit. The sample size (*n* = 77 birds per treatment; 7 replicates) was determined based on pen being the experimental unit and aligned with similar broiler breeder studies evaluating performance and egg quality traits, where 5–8 replicates per treatment are commonly used to detect biologically meaningful differences [11,12]. Each pen was fitted with one VAL-CO roaster drinker with three nipples spaced 38.1 cm apart and one Fluxx360 manual feeder (Big Dutchman, Vechta-Calveslage, Germany). Both male and female chickens received the same diets based on their treatment groups. Weekly bird body weights were used to compare target body weight curves. Two hens per pen were weighed weekly to estimate average body weight, which was used to determine feed allocation. Birds were fed once daily to ensure consistent feed intake across treatments.

### 2.4. Data Collection

#### 2.4.1. Performance Variables

Lay rate (LR) percentage was calculated as the number of eggs laid divided by the number of hens housed. The egg weight (EW) was recorded at weekly intervals by weighing all the eggs laid on the last day of the 7 days. Egg production was recorded daily by collecting all eggs from each pen within a 24 h period. Eggs were collected between 9 and 10 am every day. Pen size and hen numbers were consistent across treatments throughout the study, and the total number of eggs produced per pen was summed weekly and expressed as eggs per pen per week. Egg mass (EM) was calculated by multiplying the average EW by the total number of eggs produced in each replicate and expressed as g per hen per day. Feed conversion ratio (FCR) was calculated weekly as grams of feed consumed per gram of egg mass produced (g feed/g egg mass).

#### 2.4.2. Fertility and Hatchability (%)

Fertility and hatchability were evaluated at 30 and 41 weeks of age by collecting eggs daily over two consecutive days. A sample of approximately 12 eggs per pen was used for analysis (n = 12). Eggs were placed in a ChickMaster G18 setter (Chick Master Incubator Company, Medina, OH, USA) and the temperature was set at 37.8 °C and 58–60% humidity for 18 days. Eggs were candled on day 7 of incubation to determine fertility for each egg using an AviLighting^®^ (model AHP-ECF1, AviLighting LLC, USA). This device utilizes a high-intensity LED light source (12 V, 400 mA) designed to provide uniform illumination through the eggshell. Candling was performed in a dark room, and eggs were examined individually for fertility and embryo development characteristics, including vascularization and embryonic viability. The same operator conducted all evaluations to ensure consistency. Fertility was calculated as the total number of fertile eggs divided by the total number of eggs set for each replicate. On day 18 of incubation, fertile eggs were transferred to a Chick Master hatcher (Medina, OH, USA) set at 36.8 °C with humidity of ~65–70%. Hatchability was calculated on day 21 as the number of chicks hatched divided by the number of fertile eggs transferred to the hatchers for each hen.

#### 2.4.3. Egg Quality Variables

Egg quality parameters were assessed by collecting all eggs produced over two consecutive days at 30, 35, and 41 weeks of age. The parameters evaluated included specific gravity, egg weight, albumen height, Haugh unit, yolk color, and shell thickness. Specific gravity was quantified using the saline floatation method. Using sodium chloride (NaCl), saline solutions of densities ranging from 1.065 to 1.090 g/cm^3^ in 0.005-unit increments were prepared and stored in unique 5-gallon buckets. To prepare the saline solution, an appropriate mass of analytical-grade NaCl was gradually added to distilled water in individual 5-gallon buckets while continuously stirring to ensure a complete dissolution. The density of each saline solution was verified using a calibrated hydrometer at room temperature. Adjustments were made by adding small amounts of NaCl or distilled water until the exact target density was achieved. Once ready, each solution was labeled and stored separately for use in specific gravity measurement. For each replicate, about 13 clean, uncracked eggs were gently placed in a perforated container to allow free flow of saline solution around each egg and were eventually immersed in solutions of increasing density. The first solution in which an egg floated at the surface was recorded as its specific gravity. Egg weights were recorded using a digital bench scale (KERN FCB series; KERN and Sohn GmbH, Balingen, Germany). Each egg was cracked on an Egg Quality 3.0 Basic Equipment worktable (Bröring Technology GmbH, Lohne, Germany). Albumen height was measured using an egg-white height gauge (Bröring Technology GmbH, Lohne, Germany; Art. No. 1201) as part of the Egg Quality 3.0 system, positioned approximately 1 cm from the yolk’s edge. Yolk color was evaluated by positioning the cracked yolk over the sensor on the worktable, and the measurement was recorded via a PC connected to the device using Bröring Egg Quality 3.0 software package (Bröring Technology GmbH, Lohne, Germany), operating on a Windows 11 PC, which automatically calculated Haugh units and recorded yolk color and shell-thickness data. The eggshell thickness was measured using a digital eggshell thickness gauge (Bröring Technology GmbH, measuring range 0–10 mm, resolution 0.001 mm; Germany) by taking a small sample of eggshell from the middle portion around the equator of the egg.

### 2.5. Statistical Analysis

Data were analyzed using a completely randomized design, with pen as the experimental unit (*n* = 7 pens per treatment). Performance variables (lay rate, egg production, egg mass, and FCR) were collected weekly from 30 to 41 weeks of age and analyzed by week, with pen means used as the observational unit at each time point. Egg quality variables measured at 30, 35, and 41 weeks were analyzed separately by age. All data were analyzed using one-way ANOVA in Minitab version 22.1 (Minitab LLC, State College, PA, USA), and treatment means were separated using Fisher’s least significant difference test. Before analysis, data were checked for normality using the Shapiro–Wilk test. All data met the assumptions of normality; therefore, no data transformation was required. Statistical significance was declared at *p* ≤ 0.05, and trends were considered at 0.05 < *p* ≤ 0.10. Results are presented as least squares means ± SEM.

## 3. Results

### 3.1. Egg Quality Variables

Data on egg quality parameters, specific gravity, egg weight, shell breaking strength, yolk color, albumen height, Haugh unit, and shell thickness are shown in Table 3.

#### 3.1.1. Specific Gravity

Specific gravity was not affected by dietary treatments at any time point (Table 3). Values were similar across treatments at 30 weeks (1.0817, 1.0817, and 1.0821; *p* = 0.594), 35 weeks (1.0787–1.0788; *p* = 0.845), and 41 weeks (1.0791, 1.0795, and 1.0800; *p* = 0.644).

#### 3.1.2. Egg Weight (g)

Egg weight did not differ among treatments at 30 weeks (58.37, 58.62, and 58.82 g for control, Treatment 1, and Treatment 2; *p* = 0.619) or at 35 weeks (61.51, 61.79, and 61.66 g; *p* = 0.858). However, by 41 weeks, control hens produced heavier eggs than both Treatment 1 and Treatment 2 (*p* = 0.039), while the two treatments remained similar.

#### 3.1.3. Albumen Height (mm) and Haugh Unit

Albumen height differed among diets at 30 weeks (*p* = 0.026), with Treatment 2 showing the highest value (9.60 mm) compared to the control (9.05 mm) and Treatment 1 (9.14 mm), while Treatment 1 and control were similar. At 35 weeks, both treatments improved albumen height relative to the control (*p* = 0.018) but did not differ from each other. No differences were observed at 41 weeks (9.40–9.51 mm; *p* = 0.906).

A similar pattern was observed for the Haugh unit. At 30 weeks, T2 had the highest value (97.73), followed by T1 (96.32) and the control (95.15) (*p* = 0.029). At 35 weeks, both treatments again exceeded the control, with no difference between them. By 41 weeks, the Haugh unit did not differ among treatments (*p* = 0.618).

#### 3.1.4. Yolk Color

Yolk color scores were higher in both Treatment 1 and Treatment 2 compared to the control at all time points (*p* < 0.001). At 30 weeks, scores were 11.94 and 11.46 for Treatment 1 and Treatment 2, respectively, versus 7.83 for the control, with no difference between the treatments. By 35 weeks, all treatments differed, with Treatment 1 highest (16.15), followed by Treatment 2 (14.39) and the control (7.61). The same pattern was maintained at 41 weeks, with Treatment 1 (13.99) > Treatment 2 (11.70) > control (7.23).

#### 3.1.5. Shell Thickness (mm)

Shell thickness differed at 30 weeks (*p* = 0.034), with Treatment 1 producing thicker shells (0.377 mm) than the control (0.367 mm), while Treatment 2 was intermediate (0.372 mm) and not different from either. No differences were observed at 35 weeks (0.354–0.359 mm; *p* = 0.707). At 41 weeks, differences were not significant (*p* = 0.082), although the control showed slightly thicker shells (0.355 mm) compared to Treatment 2 (0.345 mm), with Treatment 1 remaining intermediate (0.353 mm).

### 3.2. Performance Variables

Data for laying performance, egg mass, egg production, and feed conversion ratio are shown in Table 4.

#### 3.2.1. Lay Rate (%)

Lay rate did not differ among treatments from 30 to 32 weeks (*p* ≥ 0.302) and remained similar through 40 weeks. At 40 weeks, however, Treatment 1 showed a lower lay rate (73%) compared to the control (80%) and Treatment 2 (79%) (*p* = 0.006). By 41 weeks, a trend was observed (*p* = 0.077), with Treatment 2 highest (81.4%), followed by the control (77.3%) and Treatment 1 (72.9%).

#### 3.2.2. Egg Production (Eggs/Pen/Week)

Weekly egg production was expressed as the total number of eggs produced per replicate per week. Significant differences were not observed among treatments throughout the study. At 30 weeks, egg production per replicate ranged from 58 eggs for Treatment 1 to 60.86 eggs for Treatment 2, with the Control at 58.29, but these differences were not significant (*p* = 0.345). A similar trend was observed at 31 weeks as egg production numbers were nearly identical among the 3 diets (57.71–58.86; *p* = 0.773). Egg production continued to be similar at 32 and 33 weeks (*p* = 0.806 and *p* = 0.713, respectively). Statistical differences were not observed throughout the duration of the study.

#### 3.2.3. Egg Mass (g/Bird)

Egg mass did not differ among treatments throughout the trial. At 30 weeks, values ranged from 339.11 g in Treatment 1 to 358.06 g in Treatment 2, with the control at 345.7 g (*p* = 0.432), and a similar pattern was observed at 31 weeks (350–353 g; *p* = 0.968). Although not significant, Treatment 2 was numerically higher at several time points (30, 32, 33, 35, 36, 40, and 41 weeks), peaking at 32 weeks (407.5 g), but variability was high (SEM = 32.6; *p* = 0.765). Differences approached significance at 40 weeks (*p* = 0.063), with Treatment 2 being the highest (354 g), followed by the control and Treatment 1.

#### 3.2.4. Feed Conversion Ratio (FCR) (g of Feed/g of Egg/Bird)

There were no differences in FCR among the three treatment groups from weeks 30 to 35, as indicated by *p* > 0.05. However, differences emerged in weeks 36, 40, and 41. In week 36, the Treatment 2 group achieved a 3.071 FCR, which was lower (*p* = 0.048) than the Control group (3.600) but not Treatment 1. Treatment 1 (3.171) showed intermediate values, which were not different from either the Control or Treatment 2 groups. During week 40, both the Treatment 2 (2.942) and Control (2.966) groups had significantly lower FCRs compared to the Treatment 1 group (3.228) (*p* = 0.003). In week 41, Treatment 2 exhibited the lowest FCR (2.885), which was significantly lower (*p* = 0.044) than Treatment 1 (3.171) but not Control (3.066). The Control group had an intermediate response compared to the other two treatment groups (Table 4).

#### 3.2.5. Fertility (%)

**Figure 1:**
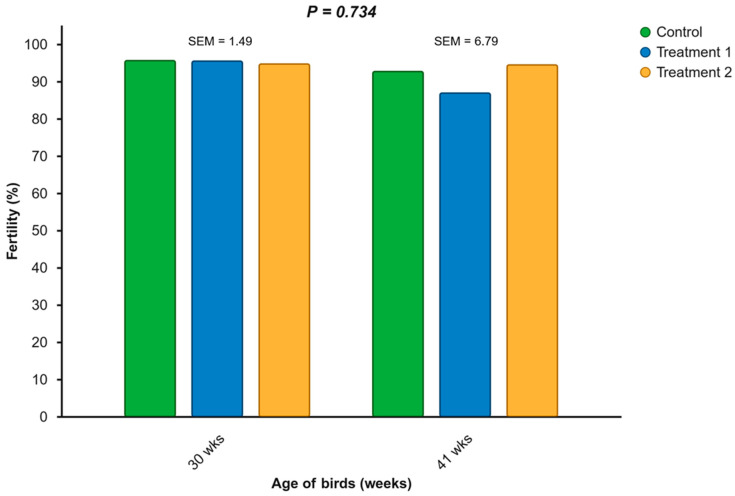
Fertility (%) of broiler breeder hens at 30 and 41 weeks of age as affected by dietary treatments. Fertility was determined as the percentage of fertile eggs relative to the total number of eggs set at each sampling age. Data are presented as least squares means ± SEM for each treatment and age. Treatment 1 = (25-OH-D_3_ and canthaxanthin), Treatment 2 = (25-OH-D_3_, canthaxanthin, copper, and gluco-oligosaccharides).

Fertility rates for the three diets, control, Treatment 1, and Treatment 2, were measured in weeks 30 and 41 (Figure 1). At 30 weeks, fertility percentages were similar among all groups: Control (96.28%), Treatment 2 (93.43%), and Treatment 1 (93.84%), with no statistically significant differences observed (*p* = 0.734). By 41 weeks, although not statistically significant, a noticeable numerical difference in fertility rates was observed among the treatments. Treatment 2 showed the highest fertility (93.6%), followed by Treatment 1 (75.6%) and Control (55.3%). Statistical analysis indicated a trend toward significance among the groups (*p* = 0.074) at week 41.

#### 3.2.6. Hatchability (%)

**Figure 2:**
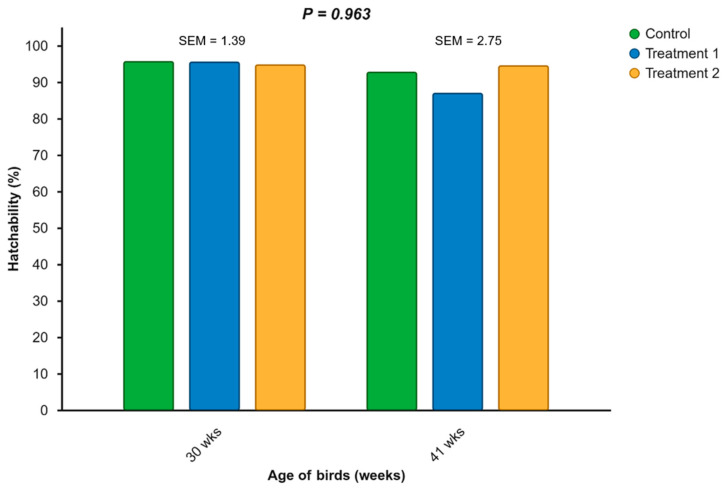
Hatchability (%) of broiler breeder eggs at 30 and 41 weeks of age as affected by dietary treatments. Hatchability was determined as the percentage of fertile eggs that hatched following standard incubation procedures. Data are presented as least squares means ± SEM for each treatment and age. Treatment 1 = (25-OH-D_3_ and canthaxanthin). Treatment 2 = (25-OH-D_3_, canthaxanthin, copper, and gluco-oligosaccharides).

Hatchability percentages for control, Treatment 1, and Treatment 2 are shown in Figure 2. At 30 weeks, hatchability was high and similar across all treatments: Control (95.57%), Treatment 2 (94.66%), and Treatment 1 (95.43%). There were no statistically significant differences among the groups (*p* = 0.963). At week 41, hatchability remained high for both the control (92.64%) and Treatment 2 (94.41%) groups, while Treatment 1 numerically showed a lower hatchability percentage (86.85%). Our results show that hatchability did not change across diets from 30 to 41 weeks. All groups maintained high hatchability rates throughout the study period, with values above 86% even in week 41.

## 4. Discussion

### 4.1. Effects of Dietary Treatments on Egg Quality

#### 4.1.1. Specific Gravity

Specific gravity was not affected by dietary treatments in the current study from 30 to 41 weeks. This could indicate that shell deposition and eggshell density were well supported by the basal diet, leaving limited room for 25-OH-D_3_, Cx, Cu, or GO to contribute to shell quality. This observation in the current study aligns with previous literature showing 25-OH-D_3_ may not always improve eggshell quality under conditions when laying hens are not vitamin D deficient, and shell quality is adequate [13]. In a long-term layer study by Chen et al. [14], eggshell variables were reported to be inconsistent across time when laying hens were fed a diet containing 25-OH-D_3_. During the early laying phase, hens exhibit high physiological performance, and eggshell-forming capacity is typically sufficient relative to later ages when shell quality often begins to decline [15]. During the early phase, the systems responsible for Ca acquisition, transport, and deposition are operating near peak efficiency. Support from vitamin D receptor signaling and active Ca transport pathways during the early phase makes Ca absorption near optimum. Medullary bone reserves are mature and easily mobilized to meet the daily Ca demand for eggshell formation [16]. The shell gland is also highly receptive with an adequate supply of carbonate and Ca transport within the uterus, enhancing mineral deposition. Coordinated activities of estrogen, progesterone, and vitamin metabolites, which constitute the endocrine regulation of eggshell formation, are highly synchronized during this period and help support shell quality [17]. As laying hens age, these mechanisms gradually become less efficient because of a decline in intestinal absorption. The late phase is also characterized by reduced medullary bone turnover and a reduced shell gland function. These factors altogether contribute to the age-related decline in shell quality. By contrast, previous literature has shown that the nutritional effects of 25-OH-D_3_ on eggshell quality are more likely to be observed in older breeders when Ca metabolism becomes less efficient, and shell quality begins to decline. A study evaluated the effects of dietary phosvitin (PV) as a potential strategy for protecting the shell and bone quality of end-of-cycle laying hens fed a Ca-reduced diet, and reported improved eggshell weight, eggshell thickness, and specific gravity [16]. In a breeder study involving vitamin D, the authors reported no difference in specific gravity in younger breeders, while inducing a difference in specific gravity at 60 weeks of age [13]. Comparing the current study to the findings from Torres et al. [13], the differences in the results for specific gravity can be attributed to the differences in the age at which specific gravity was measured, which is early phase (30 to 41 weeks) vs. late phase (60 weeks).

Prebiotics have been reported to improve gut ecology through selective stimulation of beneficial microbial populations and enhanced fermentation activity [18]. They enhance short-chain fatty acids SCFA and increase the population of beneficial bacteria without necessarily contributing to an enhancement in egg quality. For instance, a laying-hen study using chito-oligosaccharide supplementation reported no significant improvement in egg quality parameters such as eggshell strength and shell thickness [19]. Another laying-hen study involving chito-oligosaccharide supplementation reported a significant improvement in egg production and Haugh unit, but not specific gravity [20]. In contrast, Obianwuna et al. [21] reported an improvement in albumen height, Haugh unit, and eggshell thickness when 30-wk-old Hy-Line Brown laying hens were fed diets containing dietary fructooligosaccharides (FO). The contrasting response between the current study and findings from Obianwuna et al. [21] likely reflects differences in hen strain, physiological status, and dietary context. In the current study, broiler breeder hens were fed diets containing GO, while Obianwuna et al. [21] reported Hy-Line Brown laying hens’ diets containing FO.

Cu has been reported to influence metabolism through roles in enzymatic systems and antioxidant defense [22]. Specific gravity, however, is reported to be directly sensitive to Ca supply, carbonate availability, and shell gland function [23]. This means Cu may not be entirely responsible for enhancing specific gravity in laying hens. Findings from evaluating specific gravity from 30 to 41 weeks in the current study could mean that the additives, 25-OH-D3, Cx, Cu, and GO, may be biologically active, but their impact may be observed more clearly in the late production phase of broiler breeder hens. There are also chances that shell quality was good and well-regulated in the current study, and the age range of 30 to 41 weeks may be too early for additive-driven differences to be observed. This may explain the lack of differences in specific gravity observed between 30 and 41 weeks of age. In the current study, dietary inclusion of Treatment 1 or Treatment 2 did not increase egg weight at 30 and 35 weeks of age. At 41 weeks, hens on the control diets produced slightly heavier eggs than hens fed Treatment 1 and Treatment 2 (64.21 vs. 63.05 and 62.88 g; *p* = 0.039). The differences were ≈1.2–1.3 g, which is <2% of egg weight. This indicates that, under the conditions of this experiment, the control diet supported an increase in egg size during the later stages of the experiment relative to Treatment 1 and Treatment 2. This observation aligns with previous research showing that Cx − 25-OH-D_3_-based breeder supplements enhance egg quality variables such as yolk color scores, hatching rate, and progeny outcomes, but not egg weight [24]. Using Cobb 500 breeders from 25 to 62 weeks Araujo et al. [25] assessed 25-OH-D_3_ + Cx) at an inclusion rate of 1 kg/ton and reported no effects on egg weight, while yolk color scores and progeny outcomes were improved significantly. Another study in which broiler breeders were fed 6 mg/kg Cx showed increased eggshell thickness, hatchability, and chick performance with no indication of changes in egg weight [11]. In another study involving quail breeders fed different inclusion rates of Cx + 25-OH-D_3_, there was no effect on production or egg quality, but rather on the antioxidant defense system [26]. These data support the findings of the current study that a combination of Cx and 25-OH-D_3_ may not support egg weight.

#### 4.1.2. Egg Weight

The response in egg weight seen in the results aligns with other broiler breeder studies involving antioxidant additives [12,27]. When broiler breeders were supplemented with doses of vitamin E up to 100 mg/kg, there were no changes in egg weight, even though there were higher yolk color scores and an improvement in the antioxidant status of yolks [28]. The inclusion of dietary L-carnitine at levels between 200–500 mg/kg in Ross 308 from 32 to 36 weeks also did not increase egg production, egg weight, or hatchability; instead, there was a significant increase in hatch weight [12]. Other trials involving (25-OH-D_3_ + Cx) showed a contrast with the marginal depression of egg weight observed at 41 weeks by the treatments in the current study. Early to mid-lay was evaluated in Ross 708 breeder hens, whereas Araujo et al. [25] assessed Cobb 500 breeders over a longer period (25 to 62 weeks). Treatment 2 (0.05%; 25-OH-D_3_ + Cx + Cu + GO) contains extra compounds Cu and GO, which could have changed the nutrient partitioning. In some cases, parts of the absorbed amino acids and energy of the diet may have been redirected to other functions, such as immune or antioxidant defense [29]. Overall, our findings show that Treatment 1 (0.05% 25-OH-D_3_ + Cx) or Treatment 2 (0.05%; 25-OH-D_3_ + Cx + Cu + GO) did not improve egg weight in Ross 708 broiler breeders from 30 to 41 weeks. Instead, a reduction in egg size was observed at 41 weeks compared to birds receiving the control diet. The heavier eggs in the control at 41 weeks likely reflect normal biological variation and late-lay nutrient allocation toward egg size rather than egg number or efficiency; the difference, however, was marginal (<2%) and within expected variation.

#### 4.1.3. Albumen and Haugh Unit

Dietary inclusion of Treatment 1 and Treatment 2 enhanced internal egg quality as observed by higher albumen height and Haugh units at 30 and 35 weeks. However, the same was not observed at 41 weeks. Albumen height and Haugh unit were enhanced by Treatment 2 at 30 weeks relative to the control. At 35 weeks, both treatments outperformed the control by producing higher albumen height and Haugh unit. Since the Haugh unit is calculated from albumen height and egg weight and is a generally recognized indicator of albumen quality, these early responses indicate that the additive blends briefly improved the structural integrity of the albumen layer. This response is consistent with previous breeder studies in which dietary antioxidant supplementation was associated with changes in internal egg quality [27,30]. However, antioxidant status was not measured in the present study; therefore, this mechanism should be considered a possible explanation rather than a confirmed cause of the observed albumen height and Haugh unit responses. Although improvements in albumen height and Haugh unit indicate enhanced internal egg quality, these changes did not translate into measurable differences in hatchability in the present study, suggesting that the magnitude or duration of improvement may not have been sufficient to influence embryo development under early-phase production conditions. In a study where broiler breeders were fed vitamin E levels of (40–120 IU/kg), they reported an increase in Haugh unit at 35 weeks compared to the unsupplemented control [27]. This could explain why our effects lasted until 35 weeks of age. Canthaxanthin inclusion in hen diets usually results in increased yolk pigmentation instead of albumen height or Haugh unit [26]. This means that the pigments mainly target the yolk instead of the albumen proteins. The internal-quality responses observed at 30 and 35 weeks may reflect the combined effects of the additive blends, including 25-OH-D_3_, Cx, Cu, and GO, rather than the independent effect of any single component. Because Treatment 2 differed from Treatment 1 in both Cu and GO inclusion, and analyzed canthaxanthin concentration. Internal egg quality, protein, and Ca metabolism are supported by 25-OH-D3, while trace minerals may improve gut health and minimize oxidative damage to albumen proteins [30]. As laying hens get older, there is a thinning of the albumen due to aging and structural changes in the oviduct [31]. This usually supersedes the most nutritional intervention, which describes why all treatments underperformed by 41 wk despite continued supplementation.

#### 4.1.4. Yolk Color

Dietary inclusion of Treatment 1 (0.05% 25-OH-D_3_ + Cx) and Treatment 2 (0.05% 25-OH-D_3_ + Cx + Cu + GO) enhanced yolk pigmentation at the three time points (30, 35, and 41 weeks). Higher yolk color scores were observed for Treatment 1 and Treatment 2 at 30 weeks (11.49 and 11.47) vs. 7.8 for the control group (*p* < 0.001), showing a rapid deposition of Cx in the yolk. The response was more noticeable at 35 weeks as Treatment 1 recorded the highest score of yolk color based on the Roche Scale, of 16.2, followed by Treatment 2, 14.4, and then the control, 7.6. There was a decline in yolk color for all treatments at 41 weeks, but the ranking remained the same with Treatment 1 > Treatment 2 > control (*p*< 0.001), signifying Cx-enriched diets sustained darker yolk colors during the early to mid-lay period in broiler breeder hens. The result of the current study aligns with previous literature reporting that Cx is effectively absorbed from maternal diets and deposited in egg yolks. When Chinese Three-Yellow broiler breeders were fed Cx at 6 mg/kg, there was an increase in the yolk color scores compared to the control [11]. In a second study involving broiler breeder ducks, it was reported that a combination of Cx and 25-OH-D_3_ increased yolk pigmentation and the egg total antioxidant capacity (TAC) [24]. Duarte et al. [32] fed broiler breeders a Cx and 25-OH-D_3_ blend, and they reported darker egg yolks compared to the control. Studies in European quail breeders by Bonagurio et al. [26] showed similar results on yolk color with increasing dietary Cx supplementation. Treatment 1, producing higher yolk color scores at 35 and 41 weeks, might be due to the composition of the supplement mixture. Even though both Treatment 1 and Treatment 2 contain Cx and 25-OH-D_3_, Treatment 2 also contains Cu and GO. Data from Table 2 shows that, at the same inclusion level, the effective Cx concentration per kg of the diet may be slightly lower in Treatment 2 compared to Treatment 1. This could help explain why Treatment 1 showed the highest yolk color scores and most consistent pigmentation response compared to Treatment 2. From our findings, Cx reliably darkens the yolk color by increasing Cx deposition, which is in line with previous studies [26]. The consistent response of higher color scores in Treatment 1 is an indication of a more efficient pigment transfer than in treatment due to the higher pigment levels in the diet. Our research showed that relatively low inclusion levels (0.05%) of Treatment 1 and Treatment 2 are sufficient to move yolks from a basic yellow into the orange to deep-orange color range.

#### 4.1.5. Shell Thickness

In the current study, shell thickness was marginally influenced by our treatments. The effects, however, were inconsistent with the age of the birds. Hens receiving Treatment 1 at 30 weeks recorded significantly thicker shells than the control, with Treatment 2 being intermediate. The difference, however, disappeared at 35 weeks, and by 41 weeks, shell thickness values across all diets converged (Control 0.355 mm, Treatment 1 0.353 mm, Treatment 2 0.345 mm; *p* = 0.082). Previous literature supports improving shell quality using a combination of Cx and 25-OH-D_3_. In a study involving duck breeders that received a dietary supplementation of Cx and 25-OH-D_3_, it was reported that eggshell thickness was enhanced, and cracked eggs were reduced [24]. Another study involving laying hens reported that supplementing diets with 25-OH-D_3_ enhanced shell thickness through improved calcium absorption and improved mineral deposition during shell formation [33]. However, there are some studies whose findings did not support our observation for shell thickness at 30 weeks. Zhang et al. [34] assessed different dietary vitamin inclusions and reported no significant effects on egg shape index, egg specific gravity, eggshell thickness, and Haugh unit. Zhang et al. [35] in a study involving in vivo limestone solubility in Single-Comb White Leghorn hens reported that eggshell quality is highly sensitive to Ca absorption, particle size, limestone solubility, phosphorus levels, and environmental temperature.

One possible reason for the observed response on eggshell quality in our study could be due to reduced sensitivity to mineral absorption and utilization as the birds age. Hens in early lay have better intestinal absorption and shell gland function [36]. This means that the treatment diets boosted the calcium deposition by 30 and 35 weeks when the birds were younger, but at 40 weeks, when the hens were older, there was a natural decline in Ca absorption efficiency. According to [35], Ca and P requirements increase with bird age, and a normal premix may not be enough to sustain shell quality in late lay. Previous studies have indicated that adequate Ca, P, and limestone particle size affect shell mineralization, and not only vitamin D [36]. If the mineral sources in the basal diet needed additional units of Ca and P as the birds age, then this would limit the supplemented 25-OH-D_3_ or antioxidants in Cx to translate into sustained shell mineralization. The observation of eggshell quality in the current study could be due to trade-offs with other egg traits. Cu has been reported to provide trace minerals and act as a gut-health modulator [37,38,39]. It is possible that the nutrients were redistributed to support yolk pigmentation, antioxidant functions, or immune traits instead of improving eggshell thickness. There is a natural biological variation among the laying hens. The marginal differences on the order of 0.01–0.02 mm between the control and the treatment groups were numerically small.

### 4.2. Effects of Dietary Treatments on Performance

#### 4.2.1. Lay Rate

In the current study, significant differences were not observed in lay rate among diets until 40 weeks. At 40 weeks, hens in the control and Treatment 2 groups recorded relatively higher lay rates at 80% and 79%, respectively, whereas hens in Treatment 1 recorded a lower lay rate of 73%. The differences in lay rate among diets could mean that Treatment 1 and Treatment 2 did not entirely improve overall egg production from early to mid-lay but may have influenced persistence of lay at 40 weeks. The pattern observed in the current study broadly aligns with previous research in which Cx and 25-OH-D_3_ were included in breeder diets. A study in which Cx and 25-OH-D_3_ were fed to breeder ducks reported no improvement in egg production but rather reported an enhancement in yolk color, hatchability, and embryo viability [40]. In another study involving the use of Cx and 25-OH-D_3_, it was reported that, while there were improvements in yolk pigmentation, hatchability, and antioxidant status, consistent eggs-per-hen or sustained increases in lay rate were not documented [11]. Based on the data reported by previous literature, one of the reasons for the observation in the current study could be that the compounds in Treatment 1 and Treatment 2 may be more effective for enhancing egg quality rather than quantity. Zhao et al. [41] reported an improvement in laying rate when Huaixiang broiler breeders were fed dietary Cx. However, the study was conducted under high metabolic load, and there is a chance that the dietary Cx could have assisted in the mitigation of stress-induced production drop. Under stress, oxidative damage disrupts follicle development and ovulation. Canthaxanthin alleviates this damage by neutralizing free radicals, thereby maintaining ovarian performance and stabilizing lay rate [41]. In the current study, the birds were kept under standard, non-stressful conditions; the relative effects of the additives on laying rate may have been less evident. This could explain the reason a response was not observed until 40 weeks. At 40 weeks, natural production starts to decline, and antioxidant support from the Cx would become more relevant for maintaining the hens’ reproductive functions.

It could be suggested that the response observed in Treatment 2 at 40 weeks may be partly related to the inclusion of Cu and GO. However, because Treatment 2 also differed from Treatment 1 in the analyzed canthaxanthin concentration, this difference cannot be attributed solely to Cu and GO. Therefore, the finding should be interpreted as an additive response to the unique blend of the supplement rather than as the independent effect of Cu and GO. Copper is an important trace mineral that undergoes several physiological processes relevant to reproduction, including antioxidant defense, energy metabolism, and ovarian function. Copper functions as a cofactor of enzymes, including Cu or Zn-superoxide dismutase, which enhances red blood cell production and plays a role in ovarian follicle development and steroid hormone activity [22]. It has been reported that more bioavailable Cu sources can enhance metabolic efficiency, immune responsiveness under oxidative stress [42]. Even though the precise Cu levels in the Treatment 2 diet were not analytically measured in the current study, the Cu was provided through a commercial mixture intended for breeder nutrition and formulated to follow industry-accepted inclusion levels. The presence of GO in Treatment 2 could have added an extra layer of complexity through microbiome-mediated nutrient interactions. Gluco-oligosaccharides function as a prebiotic substrate, selectively stimulating beneficial microbial populations such as *Lactobacillus* and *Bifidobacterium*, which improve gut health, immune regulation, and nutrient utilization [18]. An increase in microbial biomass due to improved microbial population could have resulted in microbial sequestration or utilization of trace minerals, including Cu. Some gut microbes contain metal-binding proteins that can minimize mineral bioavailability to the host [43]. If GO accelerated microbial populations with high mineral demand, this could have reduced Cu availability and decreased its expected reproductive benefits. Microbial utilization, however, is not limited to only Cu. Prebiotic-induced changes in the microbiome may also increase microbial demand for Zn, Fe, and Mn, resulting in competitive restrictions on host nutrient availability during periods of high reproductive demand [44]. These effects would be most obvious during the late lay cycle, when nutrient partitioning becomes more limited and ovarian tissues are more sensitive to marginal deficiencies. These findings suggest that the lack of a consistent advantage of Treatment 2 could be due to an interaction between Cu supply, trace-mineral balance, and GO-driven microbiome dynamics, in which the biological benefits of Cu were partially offset by mineral competition or microbial utilization.

#### 4.2.2. Egg Production

Although the weekly egg production did not reach a statistical difference in all weeks except 40, a pattern was observed in which Treatment 2 numerically had higher numbers than the control except at 31, 34, 37, and 38 weeks (Table 4). It could be suggested that the response observed in Treatment 2 vs. the control at 40 weeks may be partly related to the inclusion of Cx, Cu and GO. These compounds have been reported to be biologically relevant in supporting the egg formation process of laying hens. 25-OH-D_3_ has been reported to enhance Ca and P metabolism, which aids eggshell formation [45]. 25-OH-D_3_ is the hydroxylated form of regular vitamin D_3_. A hydroxylated vitamin D provides the hen with the ability to immediately convert the molecule to the hormonally active form, and skip the initial conversion within the liver [46]. This process may increase the availability of vitamin D metabolites that support calcium absorption and utilization for eggshell formation [47]. Vitamin D_3_ metabolism also regulates phosphorus use by improving bone phosphorus mobilization and reducing fecal phosphorus losses, further increasing the availability of dietary phosphorus in the blood and bone tissues to support bone and eggshell formation [48]. Canthaxanthin is a fat-soluble antioxidant that is deposited in the follicles of the ovary. The follicles develop into yolks and become susceptible to oxidative stress due to the presence of large quantities of lipids. Canthaxanthin functions to protect the yolk from damage from free radicals, which cause oxidative stress. This helps to produce a healthier yolk structure, enhances nutrient integrity, and improves embryo development. This further ensures consistent ovulation and better metabolic efficiency. Copper improves egg formation in laying hens by supporting hormone production, such as Lysyl oxidase, cytochrome c oxidase, and superoxide dismutase in ovarian follicles [49]. Gluco-oligosaccharides have been reported to improve the egg-forming process by improving gut health, nutrient absorption, and boosting birds’ immune function [21]. Short-chain fatty acids that are produced from GO fermentation are potent energy molecules [21]. Short-chain fatty acids provide energy for intestinal cells. Because albumen and yolk formation require continuous protein and lipid synthesis, better energy utilization directly supports yolk deposition, albumen height, and Haugh unit [50].

#### 4.2.3. Egg Mass

In the current study, Treatment 2 consistently showed higher numerical values in 7 of the 12 weeks of evaluation for egg mass (30, 32, 33, 36, 40, and 41). Though not statistically significant, a small improvement in egg mass can lead to heavier chicks in practice. This observation could be attributed to Cu and GO, which are present only in Treatment 2. Pereira et al. [51] reported that laying hens fed trace minerals, including Cu from the early stages, showed higher oviduct weight and improved reproductive organ development. Copper is a cofactor for lysyl oxidase, which catalyzes cross-linking in collagen and elastin. This procedure is important for making the eggshell membrane, which defines the strength and integrity of the eggshell [52]. Eggshell integrity will be compromised without proper membrane formation. This may result in shell breakage and lower overall output over time. Reproductive processes such as albumen synthesis, shell, and yolk formation have been reported to produce oxidative stress [29]. Copper is a precursor for producing an important antioxidant enzyme Cu/Zn-superoxide dismutase, which functions to protect tissues such as the ovaries, the shell gland, and the liver from oxidative damage [53]. In the present study, the response observed in Treatment 2 may be related to the combined effects of the additive blend, including Cx, 25-OH-D_3_, Cu, and GO, rather than Cu and GO alone. Protecting tissues against oxidative damage sustains efficient protein and lipid synthesis and cellular integrity [54]. According to Villanueva et al. [55], laying hens fed higher Cu levels were observed to produce significantly heavier eggs, greater egg mass, and enhanced FCR. The Cu in Treatment 2 may have supported better eggshell membrane formation, thereby preventing shell-related losses. Gluco-oligosaccharides are prebiotics that act as substrates for beneficial bacteria such as Bifidobacteria and Lactobacillus. They help to improve gut integrity by increasing the villus height and absorptive surface and improve the digestion of nutrients. Obianwuna et al. [56] reported increased nutrient digestibility and improved gut integrity with FO supplementation. In situations where laying hens are faced with oxidative stress, nutrients are channeled away from egg production towards a defense mechanism. In situations like this, diets containing GO have been reported to reduce intestinal inflammation, lower systemic oxidative stress, and improve mucosal immunity [36]. There could also be a synergistic association between Cx, Cu, and GO in Treatment 2. The combination of Cx, Cu, and GO could have played a role in improving feed efficiency, antioxidant defense, and facilitating the absorption of minerals required for eggshell and membrane formation [21,57]. This is because Cx, Cu, and GO have been shown to have different physiological importance, including nutrient digestion and absorption, antioxidant defense, and eggshell formation. These physiological roles could provide a coherent understanding of the reason Treatment 2 was observed to have numerically higher egg mass values at various points in the current study.

#### 4.2.4. FCR

In the current study, Treatment 2 showed lower FCR at 36, 40, and 41 weeks, indicating the best feed efficiency. The composition of Treatment 2 contains Cu and GO, which are missing from Treatment 1 and the control. The level of Cx was lower in Treatment 2 compared to Treatment 1, suggesting a synergistic effect rather than an individual compound effect. The three compounds (Cx, Cu, and GO) have been reported to improve bird metabolism, nutrient digestion and absorption, and oxidative resilience in laying hens [8,18,57]. Copper is an important cofactor for the enzyme cytochrome c oxidase, Cu/Zn superoxide dismutase, and lysyl oxidase, which are necessary for cellular energy production, antioxidant defense, and connective tissue formation, respectively [58]. When oxidation stress is minimized, fewer nutrients are assigned towards immune defense, ovarian tissues will function adequately, and mitochondrial ATP will be stabilized [59]. This makes it possible for laying hens to allocate enough nutrients for maximum egg production while keeping intake low. Gluco-oligosaccharides enhance gut morphology and microbiota balance by increasing the villus height and villus:crypt ratio, SCFAs production, digestive enzyme function, and overall nutrient digestibility [56]. Guo et al. [60] reported that SCFAs produced from prebiotic fermentation enhance energy efficiency and reduce feed requirements for metabolic processes. The results from our study demonstrate that supplementation used in Treatment 2 may enhance feed efficiency in broiler breeders during specific weeks, particularly weeks 36, 40, and 41. The improved FCR observed in Treatment 2 suggests that the supplement may optimize nutrient utilization or metabolic efficiency during later stages of the production cycle. The lack of significant differences across the other weeks indicates that the benefits of supplementation may be time-dependent or related to specific physiological differences across the breeders’ laying cycles. It is possible that in the current study, the flock assumed a stable production plateau between 30 and 35 weeks. During this period, metabolic stress is relatively low, which could have caused ovarian follicle recruitment to be regular, yolk deposition to be efficient, and albumen secretion to be stable. Due to these changes, dietary additives may not have a measurable impact on birds’ reproduction and feed efficiency unless they are under stress. Fraser [61] reported that early-lay breeders have strong homeostatic control over reproduction, making the expression of dietary effects subtle. Treatment 1 did not provide any improvement in FCR compared to Control or Treatment 2, and at 40 weeks, it had a significantly higher FCR, suggesting that its supplementation may not provide advantages in efficiency. Unlike Treatment 2, Treatment 1 only contains the components 25-OH-D_3_ and Cx. Duarte et al. [32] reported that the inclusion of Cx and 25-OH-D_3_ in the diet of broiler breeders had a significant impact on egg production and hatchability, but not the FCR. Another study by Araujo et al. [25] involving 25-OH-D_3_ and Cx supplementation significantly improved egg production, fertility, and hatchability, but did not report an improvement in FCR. Rather, broiler chicks from breeder hens fed a combination of 25-OH-D_3_ and Cx recorded a better feed conversion ratio and higher carcass and breast yields than progeny from unsupplemented breeders when grown for 21 days [25].

#### 4.2.5. Fertility

Fertility in broiler breeders is generally high in early lay and then begins to decline progressively with advancing age, mainly due to physiological changes in both males and females [62]. Reports show that fertility usually increases from a low of 65–75% at the start of lay (23–24 weeks of age) and peaks at 95–98% at 35–37 weeks [63]. Fertility below 70% at 41 weeks is generally considered suboptimal and indicative of accelerated reproductive decline. Thus, a fertility value of 55.3% for the control at 41 weeks suggests that the hens could have experienced a more rapid reproductive decline than would typically be expected at this age. The lower fertility for the control could be attributed to physiological changes such as reduced viability of stored semen, diminished antioxidant capacity, reduced oviductal immune protection, or declining granulosa cell steroidogenesis [64]. Although fertility at 41 weeks was numerically lower in the control group than in Treatment 2, the difference was not statistically significant. Fertility was not significantly affected by dietary treatment at 30 or 41 weeks of age. Although Treatment 2 showed a large numerical fertility advantage at 41 weeks, this response should not be interpreted as evidence of a reproductive benefit because the difference was not statistically significant and the standard error was greater than 6%. Fertility was evaluated using approximately 12 eggs per pen over two consecutive days, which represented approximately 168 eggs per treatment. It would appear that this number of eggs could not overcome the pen-level variation for this variable. In a broiler breeder pen, fertility may be affected by male mating activity, semen quality, male dominance, hen receptivity, and short-term variation in mating frequency. Therefore, a large component of the numerical difference observed at 41 weeks may reflect biological and pen-level variables rather than a direct treatment effect. Future studies using more pens, multiple hatch collections within a week, and elimination of the male mating efficiency are needed to determine whether these additives influence fertility. Moreover, Treatment 2 contains 25-OH-D_3_, Cx, Cu, and GO. 25-OH-D_3_ has been reported to reduce follicular atresia and ensure the production of stronger pre-ovulatory follicles [41]. This is achieved by improving Ca transfer into the ovarian follicles, which are necessary for ensuring the structural integrity of the follicular membranes, steroid hormone production, and granulosa cell proliferation [65]. 25-OH-D_3_ improves the activities and expression of calbindin in the ovarian cells and ensures increased efficiency in Ca transfer [66]. Follicles that do not contain enough Ca cannot maintain membrane stability and are hence likely to degenerate [67]. Canthaxanthin’s antioxidative effects have been reported to protect reproductive tissues, increasing sperm and egg viability [53]. This is achieved through the antioxidant protection of reproductive cells and their membranes. Ovarian follicles and sperm cells are rich in polyunsaturated fatty acids (PUFAs), which makes them prone to lipid peroxidation [68]. Canthaxanthin is a lipophilic carotenoid that settles in cell membranes to scavenge ROS and interrupts the lipid peroxidation chain reaction [66]. This offers protection to reproductive cells and enhances sperm motility and survival, oocyte membrane integrity, and embryonic viability. Additional benefits may come from micronutrients such as vitamin E, which can support hormonal balance and immunity [69]. Vitamin E has been reported to support hormonal balance by protecting steroidogenic cells, such as granulosa and theca cells, from oxidative damage [70]. These steroidogenic cells are metabolically active and, because of this, produce a lot of ROS. Vitamin E is a lipid-soluble vitamin that functions to protect the mitochondria and preserve lipid peroxidation in steroidogenic tissues [71]. Zhang et al. [72] reported that vitamin E could positively influence hormonal balance, ensuring efficient ovulation and fertilization. Findings from Youssef et al. [73] showed that the inclusion of prebiotics in layer diets resulted in improved immune function and reproductive efficiency relative to the control birds. Prebiotics help to improve immune function by selectively stimulating the growth of beneficial bacteria such as *Lactobacillus* and *Bifidobacterium* sp. [74]. They facilitate fermentation to enhance a healthier gut by producing SCFA to support intestinal health and modulate immune responses. A review by Gündüz et al. [75] indicated that prebiotics, such as oligosaccharides, can improve mucosal immunity as well as the bird’s overall defense system, thereby enhancing resistance to pathogens and physiological stress. The inclusion of an organic Cu source in broilers showed enhanced villus height, crypt depth, an increase in beneficial gut bacteria, and a reduction in harmful bacteria [18]. These improvements in gut health can reduce enteric stress and improve nutrient absorption [56]. Copper, as a cofactor for the oxidative stress enzyme SOD, can help protect reproductive tissues against oxidative damage, which usually impairs oviduct receptivity, follicular health, and viability of embryos [76]. The numerically greater fertility observed in Treatment 2 at 41 weeks may reflect the combined effects of Cu and GO in supporting gut health, strengthening antioxidant defense mechanisms, preserving gut integrity, and facilitating nutrient absorption.

#### 4.2.6. Hatchability

None of the treatments provided a measurable advantage in hatchability compared to the control group. Hatchability remained high across treatments at both sampling ages, with values above 86% even at 41 weeks, suggesting that there was limited room for improvement under the conditions of the present study. Therefore, the current results do not provide evidence that dietary supplementation improved hatchability during the early production phase. The lack of response may also reflect the physiological stage of the birds, since breeders in early or mid-production generally have better reproductive efficiency than older breeders experiencing late-lay reproductive decline. As reported by Natsir et al. [77], laying hens in early or mid-production have optimal follicular hierarchy, sperm storage gland function, and embryo viability. Hence, the antioxidant and gut-modulating benefits of the components 25-OH-D_3_, Cx, Cu, and GO in Treatments 1 and 2 may only be fully expressed during periods of late-lay reproductive decline, oxidative stress, and high embryo mortality risk. Given that birds in the current study were assigned by body weight and fed-restricted, they were expected to receive similar feed allocations across treatments, which may limit the effects of nutritional differences on hatchability. Araujo et al. [25] reported a significant improvement in hatchability in broiler breeders fed Cx-25-OH-D_3_-based diets from 55 to 65 weeks. Their breeders were older and could be going through a period of late-lay reproductive decline. Canthaxanthin’s antioxidative effects and 25-OH-D3’s calcium benefits are more impactful when birds are physiologically challenged [26]. In the current study, hatchability data were taken at 30 and 41 weeks, during which breeder hens could be considered in a high-performing phase. Duarte et al. [62] reported that supplementing Cx and 25-OH-D_3_ improved embryo viability and hatchability, likely because the laying hens in their study were exposed to environmental stress, which enhanced the nutritional benefits of the additives. Supplementation with Cx and 25-OH-D_3_ in duck breeder diets has been shown to improve hatchability and enhance embryonic antioxidant status [40]. Differences between those findings and the present study may reflect species variation (ducks vs. chickens) and the greater oxidative challenge reported in the referenced work. Although there were no significant differences among treatments, Treatment 2 was observed to be numerically higher than Treatment 1. This could be due to the presence of Cu and GO in Treatment 2 and higher levels of Cx. Because the birds were evaluated during the early production phase, the flock may not have experienced the level of age-related reproductive decline typically observed later in lay. However, the unexpectedly low numerical fertility value in the control group at 41 weeks indicates that this interpretation should be made with caution. The fertility response at this age may also reflect pen-level variability, male mating activity, semen quality, hen receptivity, and the relatively small number of eggs evaluated per pen rather than a direct treatment effect. The numerical reduction in hatchability observed in the Treatment 1 group at week 41 may warrant further investigation, but it was not statistically significant in the current study.

## 5. Conclusions

Dietary supplementation with 25-hydroxyvitamin D_3_ and canthaxanthin, with or without copper and gluco-oligosaccharides, consistently increased yolk color, confirming effective canthaxanthin deposition in the egg yolk. Although some improvements were observed in albumen height, Haugh unit, shell thickness, lay rate, and feed conversion ratio, these responses occurred only at specific ages and were not sustained across the 30- to 41-week period. Fertility and hatchability were not significantly improved. Therefore, these additives should be interpreted as reliable enhancers of yolk pigmentation, with only limited and transient effects on selected egg quality and performance traits during early lay. Further studies over a longer production period, especially during late lay, are needed to determine whether more consistent reproductive benefits can be achieved.

## Figures and Tables

**Table 1:** Composition and calculated nutrient content of the breeder diets.

Item	Diet
Ingredients (%)	
Corn	560.40
Soybean meal	169.10
Wheat middlings	112.30
DDGS	65.00
Calcium chips	35.30
Limestone	34.02
Soybean oil	13.01
Sodium chloride	3.28
Dicalcium phosphate	2.90
DL-Methionine	1.900
Vitamin–mineral premix ^1^	1.000
L-Threonine	0.602
Sodium carbonate	0.500
Sand/test compounds ^2^	0.500
Phytase ^3^	0.208
Formulated Nutrients (%)	
Dry matter	88.37
AME (kcal/kg)	2800.00
Crude protein	15.63
Crude fat	4.09
Crude fiber	3.07
Calcium	3.00
Chloride	0.24
Available phosphorus	0.36
Total phosphorus	0.48
Potassium	0.95
Sodium	0.18
ARG	0.93
ISO	0.58
LEU	1.31
LYS	0.70
MET	0.42
MET and CYS	0.68
THR	0.60
TRP	0.18

^1^ This premix provided the following per kg of feed: 2400 IU vitamin D3, 9750 IU vitamin A, 3 mg vitamin K, 36 IU vitamin E, 14 mg DL Ca-pantothenate, 8 mg riboflavin, 0.016 mg vitamin B12, 30 mg niacin, 801 mg choline, 1 mg folic acid, 0.9 mg biotin, 3 mg thiamine, 4.5 mg pyridoxine, 70 mg manganese, 60 mg zinc, 50 mg iron, 1.25 mg iodine, 9.14 mg copper, 0.275 mg selenium, 500 mg calcium carbonate. ^2^ Control diet = 0.5 kg/MT (sand), Treatment 1 = 0.5 kg/MT (25-OH-D_3_ and canthaxanthin), Treatment 2 = 0.5 kg/MT (25-OH-D_3_ and canthaxanthin, copper, and gluco-oligosacharide). ^3^ Enzyme provided 500 FYT /kg of diet phytase.

**Table 2:** Canthaxanthin and 25-OH-D3 concentrations in experimental diets (as-is basis) used in the study.

Diets	Canthaxanthin (mg/kg)	25-OH-D_3_ (IU/kg)
Control diet		
Replication A	0	0
Replication B	0	0
Replication C	0	0
Treatment 1		
Replication A	16.6	4108
Replication B	17.4	3772
Replication C	17.5	4639
Treatment 2		
Replication A	10.4	4746
Replication B	9.2	4185
Replication C	11.1	4514

Control diet = 0.5 kg/MT (sand), Treatment 1 = 0.5 kg/MT (25-OH-D_3_ and canthaxanthin), Treatment 2 = 0.5 kg/MT (25-OH-D_3_ and canthaxanthin, copper, and gluco-oligosacharide).

**Table 3:** Effects of supplementing treatments on egg quality in broiler breeders.

Age	Control	Treatment 1	Treatment 2	SEM	*p*-Values
Specific gravity					
30 wk of age	1.0817	1.0817	1.0821	0.0003	0.594
35 wk of age	1.0787	1.0788	1.0788	0.0003	0.845
41 wk of age	1.0791	1.0800	1.0788	0.0004	0.644
Egg weight (g)					
30 wk of age	58.368	58.623	58.824	0.1916	0.619
35 wk of age	61.511	61.788	61.660	0.2016	0.858
41 wk of age	64.214 ^a^	63.050 ^b^	62.884 ^b^	0.2337	0.039
Yolk color					
30 wk of age	7.827 ^b^	11.939 ^a^	11.475 ^a^	0.1864	<0.001
35 wk of age	7.608 ^c^	16.153 ^a^	14.393 ^b^	0.2677	<0.001
41 wk of age	7.233 ^c^	13.989 ^a^	11.70 ^b^	0.1862	<0.001
Albumin height (mm)					
30 wk of age	9.050 ^b^	9.136 ^b^	9.600 ^a^	0.0899	0.026
35 wk of age	8.901 ^b^	9.578 ^a^	9.474 ^a^	0.1035	0.018
41 wk of age	9.396	9.466	9.506	0.1044	0.906
Haugh unit					
30 wk of age	95.147 ^b^	96.320 ^ab^	97.726 ^a^	0.4041	0.029
35 wk of age	94.016 ^b^	96.688 ^a^	96.715 ^a^	0.4742	0.031
41 wk of age	94.896	95.533	96.076	0.5007	0.618
Shell thickness (mm)					
30 wk of age	0.367 ^b^	0.377 ^a^	0.372 ^ab^	0.0016	0.034
35 wk of age	0.357	0.359	0.354	0.0020	0.707
41 wk of age	0.355	0.353	0.345	0.0020	0.082

^a–b^ Means within a row without a common superscript differ significantly (*p* < 0.05). Control diet = 0.5 kg/MT (sand), Treatment 1 = 0.5 kg/MT (25-OH-D_3_ and canthaxanthin), Treatment 2 = 0.5 kg/MT (25-OH-D_3_ and canthaxanthin, copper, and gluco-oligosacharide). SEM—pooled standard error of the mean (N = 7).

**Table 4:** Effects of treatment supplementation on the performance of broiler breeders.

Item	Wk30	Wk31	Wk32	Wk33	Wk34	Wk35	Wk36	Wk37	Wk38	Wk39	Wk40	Wk41
					Lay rate %							
Control	0.83	0.83	0.85	0.82	0.83	0.81	0.81	0.79	0.75	0.77	0.80 ^a^	0.773
Treatment 1	0.83	0.84	0.83	0.79	0.84	0.79	0.80	0.76	0.69	0.79	0.73 ^b^	0.729
Treatment 2	0.87	0.83	0.85	0.82	0.81	0.83	0.83	0.72	0.69	0.74	0.79 ^a^	0.814
*p*-value	0.302	0.814	0.831	0.734	0.375	0.245	0.793	0.278	0.383	0.295	0.006	0.077
SEM	0.012	0.009	0.017	0.015	0.009	0.01	0.015	0.017	0.02	0.013	0.01	0.016
	Egg production (eggs/pen/week)											
Control	58.29	58.14	59.29	57.17	58.50	56.50	54.00	52.33	49.57	50.00	53.67	50.00
Treatment 1	58.00	58.86	57.71	55.57	58.71	55.43	56.29	53.00	47.43	51.29	50.00	50.71
Treatment 2	60.86	57.71	59.57	57.57	56.57	57.43	57.29	49.86	47.29	50.57	54.00	55.00
*p*-value	0.345	0.773	0.806	0.713	0.361	0.601	0.534	0.521	0.776	0.942	0.141	0.242
SEM	0.86	0.63	1.19	1.17	1.10	1.09	1.48	1.45	1.40	1.44	1.18	1.29
	Egg mass (g/bird)											
Control	345.7	353.3	363.0	344.2	354.2	349.1	326.4	326.7	325.3	342.0	348.2	341.2
Treatment 1	339.1	351.3	348.9	329.1	358.5	342.4	353.9	323.6	298.9	327.6	323.9	329.5
Treatment 2	358.1	350.2	407.5	349.4	352.4	359.5	364.7	314.1	294.6	325.4	354.0	362.3
*p*-value	0.432	0.968	0.765	0.566	0.782	0.561	0.199	0.851	0.354	0.643	0.063	0.120
SEM	5.88	4.78	15.34	7.78	7.14	6.33	8.90	8.99	9.19	7.54	5.67	6.63
	FCR (g of feed/g of egg/bird)											
Control	3.229	3.143	3.114	3.171	3.371	3.343	3.600 ^a^	3.383	3.557	3.067	2.966 ^b^	3.066 ^ab^
Treatment 1	3.286	3.171	3.229	3.286	3.186	3.314	3.171 ^ab^	3.486	3.633	3.067	3.228 ^a^	3.171 ^a^
Treatment 2	3.086	3.171	3.186	3.086	3.329	3.186	3.071 ^b^	3.586	3.800	3.229	2.942 ^b^	2.885 ^b^
*p*-value	0.347	0.956	0.963	0.612	0.548	0.603	0.048	0.622	0.598	1.000	0.003	0.044
SEM	0.0565	0.0428	0.1628	0.0795	0.0699	0.0653	0.0958	0.1115	0.1279	0.0865	0.0647	0.0693

^a–c^ Means within a row without a common superscript differ significantly (*p* < 0.05). Control diet = 0.5 kg/MT (sand), Treatment 1= 0.5 kg/MT (25-OH-D_3_ and canthaxanthin), Treatment 2 = 0.5 kg/MT (25-OH-D_3_ and canthaxanthin, copper, and gluco-oligosacharide). SEM—pooled standard error of the mean (N = 7).

## Data Availability

The original contributions presented in this study are included in the article. Further inquiries can be directed at the corresponding author.
